# Users’ experiences with an interactive Evidence to Decision (iEtD) framework: a qualitative analysis

**DOI:** 10.1186/s12911-021-01532-8

**Published:** 2021-05-25

**Authors:** Jose Francisco Meneses-Echavez, Sarah Rosenbaum, Gabriel Rada, Signe Flottorp, Jenny Moberg, Pablo Alonso-Coello

**Affiliations:** 1 Division for Health Services, Norwegian Institute of Public Health, Oslo, Norway; 2 Epistemonikos Foundation, Santiago, Chile; 3 UC Evidence Center, Cochrane Chile Associated Center, Pontificia Universidad Católica de Chile, Santiago, Chile; 4 Norwegian Institute of Public Health, Institute of Health and Society, University of Oslo, Oslo, Norway; 5 Iberoamerican Cochrane Centre, Biomedical Research Institute (IIB Sant Pau-CIBERESP), Barcelona, Spain

**Keywords:** Decision-making, Clinical decision support, Evidence-based health care, GRADE approach

## Abstract

**Background:**

Evidence to Decision (EtD) frameworks bring clarity, structure and transparency to health care decision making. The interactive Evidence to Decision (iEtD) tool, developed in the context of the DECIDE project and published by Epistemonikos, is a stand-alone online solution for producing and using EtD frameworks. Since its development, little is known about how organizations have been using the iEtD tool and what characterizes users’ experiences with it. This missing information is necessary for any teams planning future developments of the iEtD tool.

**Methods:**

This study aimed to describe users’ experiences with the iEtD and identify main barriers and facilitators related to use. We contacted all users registered in the iEtD via email and invited people who identified themselves as having used the solution to a semi-structured interview. Audio recordings were transcribed, and one researcher conducted a directed content analysis of the interviews guided by a user experience framework. Two researchers checked the content independently for accuracy.

**Results:**

Out of 860 people contacted, 81 people replied to our introductory email (response rate 9.4%). Twenty of these had used the tool in a real scenario and were invited to an interview. We interviewed all eight users that accepted this invitation (from six countries, four continents). ‘Guideline development’ was the iEtD use scenario they most commonly identified. Most participants reported an overall positive experience, without major difficulties navigating or using the different sections. They reported having used most of the EtD framework criteria. Participants reported tailoring their frameworks, for instance by adding or deleting criteria, translating to another language, or rewording headings. Several people preferred to produce a Word version rather than working online, due to the burden of completing the framework, or lack of experience with the tool. Some reported difficulties working with the exportable formats, as they needed considerable editing.

**Conclusion:**

A very limited number of guideline developers have used the iEtD tool published by Epistemonikos since its development. Although users’ general experiences are positive, our work has identified some aspects of the tool that need improvement. Our findings could be also applied to development or improvement of other solutions for producing or using EtD frameworks.

**Supplementary Information:**

The online version contains supplementary material available at 10.1186/s12911-021-01532-8.

## Background

Decision-making in healthcare can be very complex, involving interactions between numerous actors and different kinds of information, including evidence from research [1, 2]. Although decisions about treatments, diagnostic tests, coverage, health system or public health interventions involve different types of people and different sets of information, they share a common set of factors that needs to be taken into account by groups making those decisions: the desirable and undesirable effects, values and preferences, acceptability, feasibility, and costs [3, 4] and the certainty of the available evidence. If the decision-making process is not well structured, decision makers may neglect some of these factors or give some of them too much emphasis, resulting in unbalanced judgments.

Decision making also needs to be transparent. If decisions are going to be useful, they must be communicated to target audiences (e.g., health care professionals, policy makers or patients) in an easily understandable way [5, 6]. But people may need to know more than just key messages; in order to understand the relevance of a decision for themselves or their context, they may need to know what considerations and evidence underlie a decision. A systematic approach that helps decision making groups consider all the relevant factors can facilitate a more structured and explicit process [3]; a framework documenting this process can render the decisions and underlying considerations more transparent and useful for their target audiences.

The Grading of Recommendations Assessment, Development and Evaluation (GRADE) Working Group started, in 2000, work towards the development of a systematic and transparent approach to grading quality of evidence and strength of recommendations in healthcare [7]. As part of the GRADE Working Group’s efforts, the DECIDE project—a 5-year European Union funded project aiming to improve the dissemination of evidence-based recommendations—facilitated the development of different presentations of research evidence for use in decision making, including the GRADE Evidence to Decision (EtD) frameworks [3, 4].

The GRADE-EtD frameworks help to ensure the important factors that determine a decision (the criteria) are considered and add structure to discussions. They can be used to identify reasons for disagreements in a decision-making group. Their structure can help render decisions transparent to those affected [3, 4, 7]. The frameworks include: (a) criteria, (b) judgements that must be made in relation to each criterion, (c) evidence to inform each judgement and (d) conclusions based on an overall judgement across all of the criteria [3, 4, 7]. In tackling decision making-related complexity, tailorable GRADE-EtD framework templates have been prepared for clinical practice guidelines [3], diagnostics [4], health system and public health decisions [8], and coverage decisions [9].

The iEtD is a stand-alone interactive solution for preparing and facilitating use of GRADE-EtD frameworks by decision-making groups (e.g., guidelines technical teams, panels, clinicians, and researchers). It was developed on a technical platform provided by Epistemonikos during the DECIDE project, with the aim of making it intuitive to use for people without much previous experience using the technical platform [4, 7, 10].

The iEtD provides functionality for the needs of three main groups: people preparing evidence summaries for use by decision making panels, people making decisions/recommendations, and target audiences for the decision or recommendation output. This includes functionality for creating, tailoring and editing frameworks, for individual or group voting, for documenting draft or final judgments, for exporting interim and final reports, or for reconsidering evidence, judgments and final recommendations in other settings. A central design feature is the structure, which enables placement of concise key messages from summarised evidence in close proximity to each decision criterion, making it easier for people with different levels of background knowledge to access and interpret the evidence while considering that criterion. The clear separation of criteria, evidence and judgments facilitates structured, balanced panel discussions and enhances the transparency of the final decision/recommendation. By laying the ground for a complete summary of the best available evidence to inform judgements, iEtD can facilitate both dissemination and adoption of recommendations [3, 7, 11].

Despite widespread use [4, 8, 12], little is known about current utilisation and the user experience of the GRADE-EtD frameworks, nor about the experiences of users of the online tools that include them. An evaluation of the GRADE-EtD frameworks in real guideline panels conducted by members of the GRADE group (using early paper prototypes of the frameworks) showed that methodologists and panel members perceived the frameworks as positive and useful tools that simplified the process of moving from evidence to decisions. However, participants identified some sections of the frameworks that needed improvements [13]. Panel chairs have also valued the frameworks as a useful tool for managing discussions and reaching conclusions; keeping guideline panels on track, and dealing with disagreements [7]. Since the development of the iEtD online tool for preparing and using frameworks was completed, no published studies exploring users’ experiences have been carried out. Our study was conducted to assess users’ experiences with the iEtD tool, and to identify potential tool related barriers, and factors for improvement.

## Methods

We used methods from a descriptive qualitative research [14]. We retrospectively described participants’ subjective experiences of using the iEtD tool, collecting qualitative data through semi-structured interviews. In order to analyze findings in a way that would be useful for future users and developers, we mapped data onto a framework of user experience categories and to a list of tool features, and ranked these according to their seriousness. We followed the standards for reporting qualitative research [15] (Additional file 1).

The research team was made up of members of the DECIDE project [11] that were involved in the development of both the iEtD and the GRADE-EtD frameworks (GR, PAC, JM, SR). The sum of these experiences shaped the research methods of this study, especially when it came to the design of the interview guide, and the content analysis used. In addition to the preexisting knowledge about the iEtD, we judged the research methods in this study to be fit for purpose as they are systematically developed methods previously used in other studies, including the development of the EtD frameworks [7, 16–18].

### Recruitment

We sent an introductory email to people registered in the iEtD platform, asking if they had used the tool and in what kind of context (e.g. workshop or guideline). We sent two reminders two weeks after the introductory email. We included people who were registered as users in the iEtD platform and who confirmed that they had actually used the tool, as opposed to just explored it. Once eligible participants were identified, they received an email with a brief description of the study’s aim and their potential involvement.

### Ethical considerations

All methods were carried out in accordance with relevant guidelines and regulations. Before the interviews, we informed the participants about the voluntary basis of their enrolment, which implied that they were allowed to withdraw at any time without giving a reason, and that all information collected would be used only for research purposes and treated anonymously. We did not collect any sensitive information from participants and stored their contact information separately from the data. We obtained consent to audio record sessions, and erased recordings after we finished transcribing. Transcriptions were rendered and stored anonymously.

The Regional Committee for Medical Research Ethics (REC) [19] has the authority for approving or disapproving medical research studies conducted within Norway, or by Norwegian institutions, in accordance with ACT 2009-06-20 no. 44: Act on medical and health research (the Health Research Act). This study falls outside of REC’s remit, because it is not medical or health research aiming to generate new knowledge about health and diseases. Therefore, it does not require submission for REC approval. According to REC, it therefore falls upon the Norwegian Institute of Public Health (NIPH) (employer of the lead author) to oversee that the project is carried out responsibly. NIPH does not have formalized ethical approval routines but leaves oversight up to the individual research departments. We have followed the guidelines for humanities research as laid out by the national research ethics foundation in Norway [20].

### Data collection

We organized the interviews by inviting participants to log into the iEtD and open an EtD framework, either an actual framework (e.g. from a guideline the participant was involved in) or one used for training purposes that the participant was familiar with. Then, using a semi-structured interview guide, we encouraged the participant to engage in ‘think aloud’ while exploring the tool. This is a method where a participant verbalizes their thoughts and impressions while exploring a product [21, 22]. We encouraged participants to be honest about their reactions, looked and listened for signs that they were experiencing barriers or difficulties, and posed questions when they became silent (e.g., *how was your experience with formulating the question? did you find any trouble in doing so?*). Further details are described in the interview guide (Additional file 2).

### Interview guide

We adapted an interview guide used by the DECIDE project [17, 23] that included:

1. Background questions (education, current work, previous knowledge of systematic reviews/summary of findings/GRADE)
2. Free exploration (unguided) of iEtD based on scenario text
3. iEtD walk through, one section at a time with special attention to some specific pre-determined sections that we suspected were problematic (e.g., export formats)
4. General impressions
5. Improvement suggestions

**Fig. 1:**
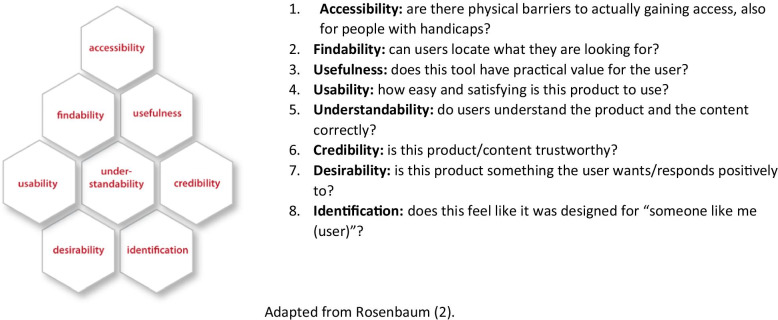
Honeycomb framework used to guide the interviews and explore users’ experiences with the iEtD. Adapted from Rosenbaum [2].

The interview guide (Additional file 2) was based on Rosenbaum’s adaptation of Morville’s “honeycomb” framework of user experience (pages 60–62, 108–116) [2]. We covered seven of the eight facets in this framework (see Fig. 1). Accessibility was not evaluated in this study, as it involves checking the application against a set of technical requirements, but can be assessed through other user experience testing methods [2]. This framework has been used and adapted based on findings from several similar studies exploring participants’ experiences of technology designed to facilitate use of research evidence in health decision making [17, 23, 24]. The framework provides a way of understanding users’ experiences of this kind of information in a way that makes direct sense to developers or designers of the information technology.

We did not set any limited time for the interviews, and the participants were always prompted to freely provide as much information as possible about their experience of use. Hence, we probed for more in-depth explanation of places where participants had problems, frustrations or were confused. Follow-up questions covered overall impressions and suggestions for improvement. Additionally, we followed a checklist to ask questions about specific pre-determined sections and functionalities of the iEtD, including presentation formats. Our main interest was to understand user’s experiences of the iEtD. Data collection involved documenting the user’s interaction with the iEtD and his/her experiences/reactions to it. With participants’ permission, we collected data in two ways:

- Audio recording (recording what the participant said while navigating through the iEtD)
- Observational notes (recording participants’ behaviour and actions, in context with what they said, and describing problems we observed). We used remote meeting software GoToMeeting (https://www.gotomeeting.com) to conduct and record the interviews.

We obtained transcripts of all interviews, and one researcher (JM) checked the accuracy and completeness of the transcripts compared to the original recordings. We based our data analysis on the interview transcriptions, informed by our observational notes.

### Data analysis

We followed a directed content approach [25] to analyze participants’ experiences in the context of the predefined iEtD structure. We chose this analytical approach due to its capability to expand conceptually the knowledge about a phenomenon that has been previously studied, but that would benefit from more in-depth research [25, 26]. Existing research shaped the analytical strategy and its coding framework.

The analysis started with a deductive content approach. Two researchers (JM and PAC) read all transcripts and highlighted data that could be mapped onto a list of features of the iEtD tool (e.g., formulating the question, assessment, or voting), which was our first coding framework [11]. This information was complemented with observational notes and exported into an Excel spread sheet. Then we categorized data according to the facets of the honeycomb framework (e.g. usability, usefulness, understandability, etc.), which constituted our second coding framework [2, 27].

Rounds of conversations between the researchers were used to ensure completeness and accuracy of the data. Furthermore, we followed an inductive content analysis of the data to uncover findings that expressed explicit or implicit need or desire for features or content that did not currently exist in the iEtD (e.g., tailoring and suggestions for improvement).

Throughout the deductive data analysis, we identified and rated findings according to the severity of the problem as expressed by the participant or observed by the researchers:

- High (show-stopper, causes incorrect interpretation, critical errors or high degree of uncertainty or dissatisfaction)
- Medium (causes much frustration or unnecessarily slow use)
- Low (minor or cosmetic problems)

We assigned codes based on our interpretation of the quotes highlighted in the deductive analysis. The codes helped us to understand in which task or location the participant experienced difficulty, and how this related to the facet(s) of the honeycomb framework when interpreting the experience of use. We also registered features that participants explicitly liked, participants’ suggestions for improvement and nice-to-haves. Finally, we sorted findings according to their severity (e.g. how much importance a finding represented for the participant’s ability to use the tool successfully), and corresponding iEtD section.

All the researchers drafted separate lists of problems users demonstrated or expressed explicitly, as well as participant suggestions for improvements. Any disagreements or discrepancies were resolved by discussion. Looking across all the findings, we searched for a more coherent way of grouping them that would be pragmatically useful for users or developers. After dialogue among project team members, we decided to organize them according to the main set of tasks that users complete when using the iEtD (e.g. tailoring and exporting frameworks).

## Results

### Participants

We contacted 860 people registered in the iEtD database. The project team sent introductory emails and two reminders in the summer of 2017. Eighty-one registrants responded to these emails (9.5% response rate), and 61 (7%) were excluded as they did not complete an entire framework for a real group decision context or for educational purposes. Twenty participants were considered iEtD users and were invited to the interviews. Eight participants agreed to be interviewed.

Participants worked in international or national organizations that developed guidelines (e.g. World Health Organization, Australian Health and Medical Research Council). Participants used the iEtD mostly for guideline development, but also for educational purposes (i.e., training workshops of panels). Two participants reported expertise in both the GRADE approach and the iEtD; two attended workshops before starting to use the iEtD, and four did not receive any training. All participants were methodologists who were members of guidelines’ technical teams, not decision-makers or panelists. Most participants had sole responsibility within their teams for completing GRADE-EtD frameworks using the iEtD solution.

### Main findings

We organized findings according to users’ general impressions of the iEtD and the tool-specific tasks users carry out using iEtD.

### Participants’ general impressions

Overall, participants had positive experiences working with the iEtD. They gave several reasons for this, such as the simplicity of the tool, that it was easy to work with, and that it was free. Users liked the way the iEtD is organized, felt that the tool was designed for someone like them, and that it was a useful tool for their organization(s). Regarding the interaction with panelists and other members of the guideline development group, they perceived the iEtD as logical and easy to follow tool during meetings:

Yes. It was really helpful both for the people compiling the evidence-to-decision framework, but also as a way [for us] to share it with the people making the decisions. So…we shared them with the guideline groups, and they used the decision-making frameworks as they were presented in this format.

Nevertheless, some drawbacks emerged from the interviews. Some participants said that due to the amount of information and type of evidence available they had to conduct additional work to synthesize and present research evidence (e.g. prepare new tables). Participants working in large groups expressed that it was difficult to coordinate framework completion work across the group.

I think the difficulty is using it in a group situation. I think you have to have a very motivated team who have all been trained in using the online version to be able to really use it well. So I think the challenge for us is that we had a big group with quite a number of different people, often from different departments, all developing their evidence profiles. So, lots of different people putting the evidence in. So, if it's a very small team I can see that it's much easier to use the online version compared to a larger team of people who may not be able to use it.

### Getting help to use the tool

Two of the participants expressed they would have liked access to online help or support; however, this did not stop them when using the iEtD. Despite being one of the least commonly used sections of the iEtD as reported by participants, some considered the *help files* as useful.

Well, first of all the little drop pin boxes that give you instructions are very helpful. So we kept referring back to those.

### Creating GRADE-EtD frameworks

#### Formulating the question and background

Participants did not report any difficulties with the PICO question section and expressed that the structure of this section was clear.

### Assessment

This section, which includes all the different criteria to be considered by a panel, was the most used section of the iEtD. However, not all teams used all of the criteria, for example when conducting rapid health technology assessments that had no formal health economic analysis. Participants’ general impressions about this section and its structure were positive. Moreover, they appreciated being able to distinguish between research evidence and additional considerations by placing them in separate cells.

Some participants criticized aspects of this section, although we observed that this was often coupled with basic misunderstandings. For instance, some participants demonstrated a poor understanding of some of the criteria (described below), the purpose of some of the features (e.g. the rationale behind Additional Considerations cells), and more fundamentally, the GRADE approach for formulating recommendations that underlies iEtD structure and functionality.

For six criteria in the assessment section (*Problem, Certainty of the evidence, Balance of effects, Resources required, Cost-effectiveness, Acceptability* and *Feasibility*), participants reported having only positive experiences. For three criteria (*Values, Desirable and undesirable effects* and *Equity*), participants had mixed experiences that we describe below.

*Values* Some participants found confusing the term “Values” (how people value outcomes) in the Assessment section menu, and others found confusing the signaling question (*Is there important uncertainty about or variability in how much people value the main outcomes?*). However, this difficulty did not stop them using the tool and no other major problems were identified.

On the ‘values’, the options are, "important”, "uncertainty" or "variability"; “possibly important”, “uncertainty or variability”; “probably not important or no important”. But the question was: “Is there important uncertainty about, or variability in how much people value the main outcome? That is a hard question, and everyone had trouble with reading it.

The way the question is phrased is the variability and how much people value it; nearly everybody had problems understanding what that means.

*Desirable and undesirable effects* Despite the overall feedback being positive, participants consistently expressed their wish to have both desirable and undesirable effects in only one section rather than in two separate sections.

*Equity* Most participants expressed favorable experiences with the use of this criterion. However, some participants referred no clear understanding of its definition:

Ah, I think we had trouble with the definition around "equity". The way that is written and defined… and how you define is... it wasn't nicely articulated so people had often difficulties with it. Otherwise, most things were reasonable.

They pointed out that there is no information about whether it refers to the intervention or the comparison, and at the time of this judgment, the panel does not know about the direction or strength of the recommendation. To solve this conundrum, two participants suggested moving recommendations right before these three criteria. Some of the comments from some of the participants reflected suboptimal understanding of the GRADE approach.

I would definitely put "recommendation" under the "desirable" and "undesirable effects". In fact, if it were up to me, I would do desirable effects, undesirable effects, and after that I would put the draft recommendation. And then I worked through values

### Conclusions section

Overall experiences with the *Conclusions* section were positive.

### Embedding tabulated summaries

Some participants found difficult to insert tables (e.g. Summary of Findings tables) to present the research evidence within the different criteria. This led them to stop using the iEtD and moved to Excel.

So it was an easy way for me to use the tool for tables, to do my own tables. And it was too much work and it was not fitting because we couldn't really... I'm trying to remember exactly what the issue was but I think the problem is that any study... So I decided to frame the table, the evidence-to-summary table as GRADE does, so starting from the outcomes. But then for the same kind of outcome we did too many different studies recording the outcomes in different ways. So even for the same kind of outcome I couldn't put anything. So eventually I decided to use the Excel.

### Use of the Export-to-Word function

The iEtD was designed to facilitate users to complete GRADE-EtD frameworks in a both online and interactive way. The tool was intended to allow people to create tailored templates for making decisions or recommendations as well as interactive end user summaries. However, such online use was not common among participants we interviewed. Many participants referred completing their work with the GRADE-EtD frameworks in a Word format rather than online. They logged on to the tool, created a framework and exported it as Word document. Overall, participants reported that other members of the guideline development group were satisfied with using the iEtD just as a guide to structure the work that then continued in Word.

So for both of those guidelines we downloaded the sheets and used them in Word format. So we used the tool as a template and that's what we used for both guideline meetings, to fill-in for quite a number of different PICO's.

But there are always people that are not confident with online tools. So I asked them, please use the Word file if you want to send me comments

The main reasons why participants preferred to work with a Word format were lack of confidence in using a new tool among members of the guideline development group, and their familiarity and perceived ease of use of Word.

It was easier to get everyone else in the team to use Word than to use it online.

People tended to find very difficult to...they were all experts in the field but they are not necessarily familiar with that sort of platform

Honestly there were also technical issues that I had to face. Not everyone is so comfortable working on these things

The non-online use of the iEtD implied extra work for the person in charge of completing the frameworks. One participant said: “I sent them, together with an instruction document explaining how to use the iEtD. Explaining what I did, what we did, and the way they would have to interpret what I did”.

### Exporting frameworks

We asked participants about their experience with the (vertical) Word document format that is generated when exporting a framework in iEtD. We also showed them another format from the GRADEpro system, which was horizontal. Participants were also asked to share their experiences with their own formats, which they had produced and tailored. Most of the participants perceived the horizontal format as clearer and more logical; they deemed the vertical format exported from the iEtD as harder to read. Moreover, participants expressed that the vertical format demanded a lot of further formatting once it was in Word:

It is repetitive; you see the same tables several times...messy

It is not friendly,…., and requires too much editing to be able to generate a document that is easily usable and readable by decision makers

I think, while the information is the same it doesn't feel like I can see things so well but I think is just because it's all... it's feels like it's more text, which is rare because it's the same text, but it's not as appealing to me.

### Tailoring frameworks

Some participants tailored the frameworks. It was common for people to translate and modify the wording, particularly of the judgment options.

I think it was felt that it was too... introduced too much uncertainty, to have the options as they are... some of them we took out the "various" option, so that we just had, "don't know", "no", "probably no", "probably yes", "yes".

Participants viewed tailoring as a valuable functionality. It gave them the possibility to modify the frameworks to their specific needs, such as limiting the number of criteria for rapid health technology assessments or modifying the order of the criteria to improve understanding.

### Motivations to use the iEtD

Despite some difficulties, participants still expressed motivation to use the iEtD. Some chose it because it is part of the GRADE approach, and they were familiar with it. The attendance to iEtD workshops was also highlighted as a facilitator. Participants said that the systematic and comprehensive structure of the iEtD was a crucial aspect for deciding to use it. They considered it a suitable tool for producing systematic and transparent guidelines, as it provided a comprehensive overview of the different factors involved. Most of the participants expressed that they would like to receive further training on the tool.

We decided to take the iEtD because we it was a good match between the dimensions considered in framework, to assess the effectiveness and feasibility

I went through the criteria for the evidence-to-decision framework and I found that it fit quite well with what I was looking for, a kind of framework or methodological system that could allow me to include everything. So criteria-like values, equity, feasibility, acceptability, were all criteria that we were considering in our guidelines. So that was eventually the reason....

In addition, participants expressed they chose the iEtD partly due to the online voting function that could be used during meetings.

Well, we wanted to do real time voting with in the panel meeting and so because that feature was available, and because it was easy to migrate from MAGIC into this, we decided to go with it

### Using iEtD in guideline meetings

In the context of guideline meetings, voting was one of the features most valued by participants and received positive feedback from most. However, the ways that groups used the voting function varied. For instance, some collected votes manually outside the iEtD, then compiled results and entered them into the system.

We did the voting two ways. We started by asking each panel member to go in and register their vote and comment, and that provided a baseline. We extracted all that information and circulated it to the whole group. Then, we met and put the information up on the screen –and did it live–and we read through and amended it, and then we all voted.

…only one person in the room had the iEtD framework opened, projected on the screen, and counted out the votes and recorded them in the iEtD.

A few participants expressed connectivity issues when working online.

The system could not take all ten of us working on the same iEtD, at the same time voting in the same way, so we stopped doing that

When we used it live, when everyone was online at the same time and they were all voting together it kept crashing, so what we actually moved into was... we printed the relevant document note, extracted the relevant document, and tables and headings, and send them to people on an email, and they completed the framework. They send it back to us

### Participants’ suggestions

Two main suggestions for improvement emerged from the interviews: (1) need to provide more guidance, including examples, about what type of information should be include in each of the criteria; (2) need to improve the wording of some domain headings, signaling questions as well as more detailed definitions. We compiled a list of problems and potential suggestions for further improvement of the iEtD tool (Table 1).

**Table 1:** Participants’ suggestions and potential solutions

Suggestions	Potential solutions
Preference for different order and number of sections and criteria	Making explicit the already available possibility of collapsing several criteria Make possible to change the order of sections and criteria (e.g., the recommendation stands at the top rather than at the bottom)
Large amount of work and time invested when completing frameworks in the iEtD	Raise awareness regarding the iEtD's flexibility (e.g., that it is not mandatory to include all criteria or to prepare systematic reviews for each criterion). The amount of work needs to be tailored to the resources of each organization
Difficulties when working with large groups	Small technical teams might be optimal size; explore what kind of extra guidance might be needed for larger technical teams Make sure good Internet connectivity is available for the work with large groups Rigorous technical testing needed, simulating use by large panels to resolve stability issues
Unclear wording of terminology and of the signaling questions in the Assessment section	Improve guidance, both general and contextual Improve wording of the criteria. For example, specify for equity whether it refers to the intervention or the comparison Increase training possibilities. For example, providing tutorials or related resources
Difficulties when inserting Summary of Findings (SoF) tables	Possible integration with GRADE-Pro or to facilitate a more flexible way of including tables in the iEtD Further training on how to use the iEtD and other resources, such as Interactive Summary of Findings (iSoF) tables
Preference of some users for the horizontal presentation format (rather than vertical) of the cells for each criterion	Include an additional horizontal presentation format, both for visualization and for its exportation

## Discussion

### Main findings

Our study explored users’ experiences with the iEtD tool in real scenarios. The majority of participants reported an overall positive experience, without major difficulties navigating or using the different sections. They also reported having used most of the framework criteria satisfactorily. Participants reported tailoring the frameworks, for instance by adding or deleting criteria, translating to another language, or by rewording headings. Some participants reported concerns with the having to edit the exportable formats.

We also uncovered some difficulties people experienced using the iEtD. The tool did not work well in large panels, probably due to the panelists’ lack of familiarity with the tool as well as some technical instability. A more structured process and group dynamics could facilitate a more favorable experience. A non-online use of the iEtD was also common in this study, as participants preferred to export frameworks to a Word format and then work with them on paper. Findings revealed that panels had highly varying workflows, technical environments, and degrees of familiarity with the tool; this demonstrates the importance of building tools that are highly flexible in use, a finding with implications for other EtD interactive technologies. But it also underscores that many technical teams prefer to complete their work in a more standard format. Improving export formats would help those users who prefer to work in Word.

### Our results in the context of previous research

Consistent with the findings of the development process of the iEtD [7], participants in our study found the iEtD intuitive and easy to work with. Our participants also highlighted the framework structure as a positive factor for facilitating structured discussions among panelists. Li and colleagues, recently reported similar experiences with the use of the GRADE-EtD frameworks in face-to-face panel meeting discussions for guidelines for the management of venous thromboembolism [12]. In their study, authors found that the frameworks not only provided structure but also ensured that the panelists considered all relevant criteria for making decisions. Guideline panelists also experienced the use of the GRADE-EtD frameworks as to be more straightforward when the evidence available was sufficient and clear [12], In a study about the development of WHO guidelines for task shifting, authors describe valuing use of EtD frameworks to structure discussions about a large and complex body of evidence, including qualitative evidence. However, although they valued expanding the evidence base for decision making, they found that summarizing evidence for multiple criteria was very resource demanding [28]. Likewise, we observed that some users complained about the magnitude of the work involved completing the framework, given the number of criteria included, the implied expectation that they needed to provide evidence for all criteria, and the difficulty of creating succinct evidence summaries.

### Limitations and strengths

We carried out in-depth interviews with eight people, providing us with rich data about their experiences with iEtD that helped us identify several significant problems they had. However, during the interviews, we discovered that the participants varied much more regarding familiarity with the tool and the GRADE approach than we anticipated. Earlier research gives us reason to believe that degree of familiarity with the GRADE approach will impact people’s experience of the EtD frameworks [7]. Therefore, we can’t be confident that we have uncovered all the main problems, either for people who are familiar with GRADE, or likewise, for people who are less familiar with GRADE. Our study would likely have been strengthened by focusing on a more homogenous group of participants.

This study exhibits some limitations inherent to both the methodological design and its analytical strategy. Following a directed content analysis means that researchers will approach the data with preconceived ideas about the phenomenon of interest [25, 26]. This makes them more prone to find and communicate findings that are supportive rather than non-supportive for those previous ideas. This limitation could be reinforced by an overemphasis in the predefined categories of the coding frameworks that guided the analysis. Additionally, some participants may have provided feedback intended to please the researchers. Furthermore, and despite the flexibility of the research methods used, the deductive analysis preceded the inductive analysis, which could have constrained the possibilities of identifying additional, unexpected insights from the participants about their experience of use.

A potential additional limitation is that the majority of the authors were involved in the development of the iEtD. However, the iEtD is an expert tool, and it would not be possible to fully comprehend participants’ user experiences without having a comprehensive prior understanding of how the tool works and what it can do. None of the members of the had any previous professional relationship with the participants interviewed.

To the best of our knowledge, this is the first study addressing the technical teams’ user experiences with the use of electronic EtD frameworks. Our findings are applicable for both methodologists and technical team members of guideline development groups who have experience in completing GRADE-EtD frameworks in real scenarios. We followed research rigorous methods that have been used in previous studies in the field of evidence-informed decision-making [7, 13, 17, 23]. Time from actual use to the time of the interview ranged from 3 years in one participant to a couple of months in two of them. The interview guide helped us ensure a comprehensive exploration of the iEtD and facilitated appropriate recall by participants. Nevertheless, we do not discard the likelihood of recall bias.

### Implications for practice and research

Guideline developers may use our findings to improve their experiences with the iEtD, for instance by using a trained technical team, helping them develop skills to create succinct evidence summaries, and providing training for panels, both in the GRADE approach and the GRADE-EtD frameworks. Panels using the iEtD and EtD frameworks in general will benefit from the use of a highly structured process and optimal group dynamics. Developers of other online tools that include the GRADE-EtD frameworks may also find our results and suggestions for improvement valuable.

In this study, we identified important facilitators of a positive user experience. Further research will help a better understanding in the use of online tools for evidence-informed decision-making processes. Findings from our study might serve as a starting point to explore the extent to which guideline development groups and panelists discuss and use multi-criteria frameworks for decision-making processes. Further research is also needed on which are the factors that influence the use of online tools for decision making, such as group composition, communication styles, and contextual/situational factors. Various methods can be used in further research, such as participatory and non-participatory observation of guideline panels and workshops, prototype sketching, testing examples, user-test interviews, stakeholder feedback, surveys, questionnaires, and discussions in face-to-face meetings [7]. Further research would also benefit from real world testing of the iEtD, for instance with technical teams who are familiar with the EtD frameworks but not necessarily with the iEtD. Finally, we also recommend exploring experiences of others beyond technical teams, such as panel members, chairs, and end users of the different presentation formats.

## Conclusions

A very limited number of guideline developers have used the iEtD tool published by Epistemonikos since its development. Although users’ general experiences are positive, our work has identified some aspects of the tool that need improvement. Our findings could be also applied to development or improvement of other solutions for producing or using EtD frameworks.

## Supplementary Information

**Additional file 1**.

**Additional file 2**.

## Data Availability

Not applicable.

## Abbreviations

GRADE approach: The Grading of Recommendations Assessment, Development and Evaluation (GRADE) approach
GRADE-EtD frameworks: Evidence to Decisions frameworks
GRADEPro GDT: The GRADEPro Guideline Development Tool
iEtD: Interactive Evidence-to-Decision

## References

[1] Lavis JN, Ross SE, Hurley JE, Hohenadel JM, Stoddart GL, Woodward CA (2002). Examining the role of health services research in public policymaking. Milbank Q.

[2] Rosenbaum SE (2010). Improving the user experience of evidence. A design approach to evidence-informed health care.

[3] Alonso-Coello P, Schünemann HJ, Moberg J, Brignardello-Petersen R, Akl EA, Davoli M (2016). GRADE Evidence to Decision (EtD) frameworks: a systematic and transparent approach to making well informed healthcare choices. 1: Introduction. BMJ.

[4] Schunemann HJ, Mustafa R, Brozek J, Santesso N, Alonso-Coello P, Guyatt G (2016). GRADE Guidelines: 16. GRADE evidence to decision frameworks for tests in clinical practice and public health. J Clin Epidemiol.

[5] Grimshaw JM, Eccles MP, Lavis JN, Hill SJ, Squires JE (2012). Knowledge translation of research findings. Implement Sci IS.

[6] Liang Z, Howard PF, Leggat SG, Murphy G (2012). A framework to improve evidence-informed decision-making in health service management. Aust Health Rev Publ Aust Hosp Assoc.

[7] Rosenbaum SE, Moberg J, Glenton C, Schünemann HJ, Lewin S, Akl E (2018). Developing evidence to decision frameworks and an interactive evidence to decision tool for making and using decisions and recommendations in health care. Glob Chall.

[8] Moberg J, Oxman AD, Rosenbaum S, Schunemann HJ, Guyatt G, Flottorp S (2018). The GRADE Evidence to Decision (EtD) framework for health system and public health decisions. Health Res Policy Syst.

[9] Parmelli E, Amato L, Oxman AD, Alonso-Coello P, Brunetti M, Moberg J (2017). GRADE Evidence to Decision (EtD) framework for coverage decisions. Int J Technol Assess Health Care.

[10] Alonso-Coello P, Oxman AD, Moberg J, Brignardello-Petersen R, Akl EA, Davoli M (2016). GRADE Evidence to Decision (EtD) frameworks: a systematic and transparent approach to making well informed healthcare choices. 2: clinical practice guidelines. BMJ.

[11] DECIDE 2011–2015. Interactive GRADE Evidence to Decision (iEtD) framework [cited]. Available from http://www.decide-collaboration.eu/interactive-evidence-decision-ietd-framework.

[12] Li SA, Alexander PE, Reljic T, Cuker A, Nieuwlaat R, Wiercioch W (2018). Evidence to Decision framework provides a structured "roadmap" for making GRADE guidelines recommendations. J Clin Epidemiol.

[13] Neumann I, Brignardello-Petersen R, Wiercioch W, Carrasco-Labra A, Cuello C, Akl E (2016). The GRADE evidence-to-decision framework: a report of its testing and application in 15 international guideline panels. Implement Sci IS.

[14] Sandelowski M (2000). Whatever happened to qualitative description?. Res Nurs Health.

[15] O'Brien BC, Harris IB, Beckman TJ, Reed DA, Cook DA (2014). Standards for reporting qualitative research: a synthesis of recommendations. Acad Med.

[16] Rosenbaum SE, Glenton C, Cracknell J (2008). User experiences of evidence-based online resources for health professionals: user testing of The Cochrane Library. BMC Med Inform Decis Mak.

[17] Rosenbaum SE, Glenton C, Nylund HK, Oxman AD (2010). User testing and stakeholder feedback contributed to the development of understandable and useful Summary of Findings tables for Cochrane reviews. J Clin Epidemiol.

[18] Rosenbaum SE, Glenton C, Oxman AD (2010). Summary-of-findings tables in Cochrane reviews improved understanding and rapid retrieval of key information. J Clin Epidemiol.

[19] REK: Norwegian Committees for Medical and Health Research Ethics. Examples of activities that do not require approval from REC. Oslo [cited]. Available from https://helseforskning.etikkom.no/reglerogrutiner/soknadsplikt/sokerikkerek?p_dim=34999&_ikbLanguageCode=us.

[20] The National Committee for Research Ethics in the Social Sciences and the Humanities (NESH). Guidelines for Research Ethics in the Social Sciences, Humanities, Law and Theology [cited]. Available from: https://www.forskningsetikk.no/en/guidelines/social-sciences-humanities-law-and-theology/guidelines-for-research-ethics-in-the-social-sciences-humanities-law-and-theology/.

[21] Jaspers MWM, Steen T, Bos C, Geenen M (2004). The think aloud method: a guide to user interface design. Int J Med Inform.

[22] Eccles DW, Arsal G (2017). The think aloud method: what is it and how do I use it?. Qual Res Sport Exerc Health.

[23] Rosenbaum SE, Glenton C, Cracknell J (2008). User experiences of evidence-based online resources for health professionals: user testing of The Cochrane Library. BMC Med Inform Decis Mak.

[24] Rosenbaum SE, Glenton C, Wiysonge CS, Abalos E, Mignini L, Young T (2011). Evidence summaries tailored to health policy-makers in low- and middle-income countries. Bull World Health Organ.

[25] Hsieh H-F, Shannon SE (2005). Three approaches to qualitative content analysis. Qual Health Res.

[26] Assarroudi A, Heshmati Nabavi F, Armat MR, Ebadi A, Vaismoradi M (2018). Directed qualitative content analysis: the description and elaboration of its underpinning methods and data analysis process. J Res Nurs.

[27] US Dept of Health & Human services User Experience Basics. [Cited]. Available from https://www.usability.gov/what-and-why/user-experience.html.

[28] Glenton C, Lewin S, Gülmezoglu AM (2016). Expanding the evidence base for global recommendations on health systems: strengths and challenges of the OptimizeMNH guidance process. Implement Sci.

