# Correction to: Users’ experiences with an interactive Evidence to Decision (iEtD) framework: a qualitative analysis

**DOI:** 10.1186/s12911-021-01559-x

**Published:** 2021-07-07

**Authors:** Jose Francisco Meneses-Echavez, Sarah Rosenbaum, Gabriel Rada, Signe Flottorp, Jenny Moberg, Pablo Alonso-Coello

**Affiliations:** 1 Division for Health Services, Norwegian Institute of Public Health, Oslo, Norway; 2 Epistemonikos Foundation, Santiago, Chile; 3 UC Evidence Center, Cochrane Chile Associated Center, Pontificia Universidad Católica de Chile, Santiago, Chile; 4 Institute of Health and Society, University of Oslo, Oslo, Norway; 5 Iberoamerican Cochrane Centre, Biomedical Research Institute (IIB Sant Pau-CIBERESP), Barcelona, Spain

## Correction to: BMC Med Inform Decis Mak (2021) 21:169 https://doi.org/10.1186/s12911-021-01532-8

Following publication of the original article [1], it was reported that there was an error in affiliation 4.

The incorrect version was:

Norwegian Institute of Public Health, Institute of Health and Society, University of Oslo, Oslo, Norway.

The correct version is:

Institute of Health and Society, University of Oslo, Oslo, Norway.

The original article has been updated.
