# Supplementary material for: Users’ experiences with an interactive Evidence to Decision (iEtD) framework: a qualitative analysis
Source: BMC Med Inform Decis Mak. 2021 May 25;21:169. doi: 10.1186/s12911-021-01532-8 (PMC8146986; doi:10.1186/s12911-021-01532-8)
Supplement: Supplementary file 2 — Additional file 2. [file 12911_2021_1532_MOESM2_ESM.docx]

**Users’ experiences with an interactive Evidence to Decision (iEtD) framework: a qualitative analysis**

**Additional file 2. Interview guide:** **Interactive Evidence to Decision framework (iEtD)**

| **Users’ experiences of the Interactive Evidence to Decision framework (iEtD): Interview guide** | |
| --- | --- |
| **Test person no.:** |  |
| **Scenario example:** |  |
| Place: |  |
| Date: |  |
| Interviewer/note taker: |  |

**BACKGROUND – ABOUT PARTICIPANT AND PHASE OF FAMILIARITY/USE**

**Who/what?**

1. Who are you representing?
2. Are you representing an **organization** or did you explore iEtD by yourself?

____________________________________________________________________________________________

1. What is the actual or past **context** of use? (What kind of guidelines/decisions, for whom?) Please describe it: _______________________________________________________________________________
2. IF RELEVANT: what were the main **reasons for NOT using** the iEtD at present?

____________________________________________________________________________________________

*Prompts*: “The organization was short of staff, no time, lack of IT resources/support, no funding, there was no organizational support/incentive”. Look/explore into the following levels:

- **Structural and external levels**: conditions/factors from outside the organization.
- **Organizational level** (e.g., leadership, inadequate financial investments, lack of policy and political will, infrastructure, readiness).
- **User level**: issues related to user characteristics (for instance attitude, motivation, skill, will).

- **Tool level**: characteristics of iEtD itself.

Notes: **____________________________________________________________________________________________**

**What phase – pre-adoption or adoption?**

1. How much you have explored/used it (depth of use, frequency of use, etc)?

____________________________________________________________________________________________

1. What is your current use status?

- EXPLORATION: have only used iEtD so far, making an account in iEtD, exploring an example, exploring with own content, presenting to colleagues/management for consideration of use, organization performed an evaluation to inform decision about use…

- USE: actual or past context of use in one or more guideline/decision contexts, or in the process of establishing/sustaining routine use in their context.

**Moving from exploration to use

Any tailoring?**

1. IF RELEVANT: Did you (or your organisation/a colleague) tailor iEtD somehow (e.g., create own templates? What did you add/change? Translate templates to another language)? Describe type of tailoring and any problems or feedback related to that.

____________________________________________________________________________________________

Prompt: If they have not tailored or are unaware of this function, ask whether they find it useful

**Training or support?**

1. Did you receive any instruction from anybody to get started?
2. Did you find or access any of the help files/sections? (i.e., methodological help or functional help?) Or did you search unsuccessfully for help?
3. What kind of support or instruction did you need/do you currently need to move on?

____________________________________________________________________________________________

**Barriers/facilitators**

1. If you are using iEtD, what are the main reasons?

Please tell us about what factors/situations **led you to use iEtD**: ____________________________________________________________________________________________

**FREE EXPLORATION OF THE iEtD**

This section will be done **only with those users who have completed a framework in iEtD** and that feel confident enough with the tool. Users will be invited to open the framework they created and point out the functionalities where they experienced troubles.

**WALK-THROUGH**

We will walk all participants, those that participated in the free exploration, through the main sections and functionalities of the iEtD following a checklist (see below). For those that conducted a free exploration we will go through the sections that they did not comment upon during the exploration.

We will go through each major section by following the checklist below, and prompt the user to point out the functionalities if they used it and whether they experienced any difficulties.

| **Note:** Keep these questions on a separate sheet for yourself during the interview, and use them to probe more in-depth about areas where people report difficulties.  When they indicate that they experienced difficulties, we will elicit more information about those difficulties using the following probes:   - Description of the problem - Severity of the Problem (1 = Trivial to 5=Critical) - Where the user encountered the problem (URL or screen) - What the user was doing (which task) - Expectation about what should have happened - Could the user recover? If so how? - (Possible design/programming solutions to the problem) |
| --- |

**Checklist for the walk through:**

**[ ] List page**

**[ ] Question**

[ ] Did you use this section? [ ] Yes [ ] No

[ ] If so, which parts did you have difficulties with?

**____________________________________________________________________________________________**

**[ ] Assessment**

[ ] Did you use this section? [ ] Yes [ ] No

[ ] If so, which parts did you have difficulties with?

**____________________________________________________________________________________________**

[ ] Note: Show the structure of one of the criteria (Research Evidence, Additional Considerations, Judgments, Detail Judgments, Panel Discussion) and ask whether they found it useful/easy to use? Any difficulties?

**____________________________________________________________________________________________**

[ ] Did you use the **voting functionality**? How, and did you find it useful?

**____________________________________________________________________________________________**

[ ] Show them the criteria and ask if they used them all. If not ask them why and if they experienced any difficulties or have comments about any of the criteria.

| **Assessment** | Used it | Difficulties |
| --- | --- | --- |
| Problem | [ ] | [ ] |
| [Text to insert notes] | | |
| Desirable effects | [ ] | [ ] |
| [Text to insert notes] | | |
| Undesirable effects | [ ] | [ ] |
| [Text to insert notes] | | |
| Certainty of the evidence | [ ] | [ ] |
| [Text to insert notes] | | |
| Values | [ ] | [ ] |
| [Text to insert notes] | | |
| Balance of effects | [ ] | [ ] |
| [Text to insert notes] | | |
| Resources required | [ ] | [ ] |
| [Text to insert notes] | | |
| Certainty of evidence of required resources | [ ] | [ ] |
| [Text to insert notes] | | |
| Cost-effectiveness | [ ] | [ ] |
| [Text to insert notes] | | |
| Equity | [ ] | [ ] |
| [Text to insert notes] | | |
| Acceptability | [ ] | [ ] |
| [Text to insert notes] | | |
| Feasibility | [ ] | [ ] |
| [Text to insert notes] | | |

| **Conclusions** | | |
| --- | --- | --- |
| Summary of judgments | Used it | Difficulties |
|  | [ ] | [ ] |
| [Text to insert notes] | | |
| Type of recommendation | [ ] | [ ] |
| [Text to insert notes] | | |
| Recommendation | [ ] | [ ] |
| [Text to insert notes] | | |
| Justification | [ ] | [ ] |
| [Text to insert notes] | | |
| Subgroup considerations | [ ] | [ ] |
| [Text to insert notes] | | |
| Implementation considerations | [ ] | [ ] |
| [Text to insert notes] | | |
| Monitoring and evaluation | [ ] | [ ] |
| [Text to insert notes] | | |
| Research priorities | [ ] | [ ] |
| [Text to insert notes] | | |

[ ] **Rest of the sections in the left hand menu** (i.e., evidence profile, references, footnotes,

glossary, conflicts of interest and/or appendices)?

| [Insert section] | Used it  [ ] | Difficulties  [ ] |
| --- | --- | --- |
| [Text to insert notes] | | |
| [Insert section] | | |
| [Text to insert notes] | | |

| Toolbar functionality (i.e., guidance for sections, expand/collapse fields, adding comments, switch to view suitable for presentations, and creating presentations) | Used it  [ ] | Difficulties  [ ] |
| --- | --- | --- |
| [Text to insert notes] | | |
| [Insert section/notes] | | |
| [Text to insert notes] | | |

**Notes:** _____________________________________________________________________________________________

**Other questions to keep in mind:**

- Did you like how the framework is organized?

- How comfortable are you with using the menu?

- What applications/functions do you use most often?

**SPECIFIC QUESTIONS:**

**Did you use any of the presentation formats?** [ ] Yes [ ] No

[ ] If so, which formats have you used?

**____________________________________________________________________________________________**

[ ] If so, which ones did you have difficulties with?

**____________________________________________________________________________________________**

1. **Exportable formats**

a) Facilitate the Word format downloaded from iEtD.

b) Ask about user’s impressions.

*Prompts: Is it useful/friendly?* *is the format easy to download? Is all the key information contained into the exportable format?*

____________________________________________________________________________________________

a. Show the download format from **GRADEpro-GDT**

b. Ask about user’s impressions.

*Prompts: Is all the key information contained into the exportable format? Is it useful/friendly?*

Which exportable format did you find particularly useful?

**____________________________________________________________________________________________**

Ask specifically about the Summary of judgments. Used it? Useful?

**____________________________________________________________________________________________**

**GENERAL IMPRESSIONS**

1. Can you comment on the degree to which the tool is useful or not, for you, your work or your organization (and give a brief explanation for your answer)?
2. Do you feel that this tool is designed specifically for use by someone like you (or your organization) or not?
3. Can you summarize your general impressions of iEtD tool, both positive and negative?

____________________________________________________________________________________________

**SUGGESTIONS – Improvements on iEtD**

1. Do you have any suggestions for changes that would make iEtD a better tool for his/her purposes?

**Only in the case** their organization was initially interested but discontinued use or didn’t move forward, what would need to happen in order for them to start using the tool? ____________________________________________________________________________________________

This was all. Thank you for your time and collaboration! **End**
